# Fast automated reconstruction of genome-scale metabolic models for microbial species and communities

**DOI:** 10.1093/nar/gky537

**Published:** 2018-06-21

**Authors:** Daniel Machado, Sergej Andrejev, Melanie Tramontano, Kiran Raosaheb Patil

**Affiliations:** European Molecular Biology Laboratory (EMBL), Meyerhofstrasse 1, 69117 Heidelberg, Germany

## Abstract

Genome-scale metabolic models are instrumental in uncovering operating principles of cellular metabolism, for model-guided re-engineering, and unraveling cross-feeding in microbial communities. Yet, the application of genome-scale models, especially to microbial communities, is lagging behind the availability of sequenced genomes. This is largely due to the time-consuming steps of manual curation required to obtain good quality models. Here, we present an automated tool, CarveMe, for reconstruction of species and community level metabolic models. We introduce the concept of a universal model, which is manually curated and simulation ready. Starting with this universal model and annotated genome sequences, CarveMe uses a top-down approach to build single-species and community models in a fast and scalable manner. We show that CarveMe models perform closely to manually curated models in reproducing experimental phenotypes (substrate utilization and gene essentiality). Additionally, we build a collection of 74 models for human gut bacteria and test their ability to reproduce growth on a set of experimentally defined media. Finally, we create a database of 5587 bacterial models and demonstrate its potential for fast generation of microbial community models. Overall, CarveMe provides an open-source and user-friendly tool towards broadening the use of metabolic modeling in studying microbial species and communities.

## INTRODUCTION

Linking the metabolic phenotype of an organism to environmental and genetic perturbations is central to several basic and applied research questions. To this end, genome-scale metabolic models provide a mechanistic basis allowing to predict the effects of, e.g. gene knockouts, or nutritional changes (1,2). Indeed, such models are currently used in a wide range of applications, including rational strain design for industrial biochemical production (3,4), drug discovery for pathogenic microbes (5), and the study of diseases with associated metabolic traits (6–9).

An emerging application of genome-scale models is the study of cross-feeding and nutrient competition in microbial communities (10–15). However, a vast majority of relevant microbial communities, such as those residing in the human microbiota (16), ocean (17) or soil (18), still remain inaccessible for metabolic modeling due to the unavailability of the corresponding species-level models. Thus, applications of metabolic modeling lag behind the opportunities presented by the increasing number of genomics and metagenomics datasets (19). A major bottleneck is the so-called genome-scale reconstruction process, which often requires laborious and time-consuming curation, without which the model quality remains low. This becomes an even more stringent bottleneck considering that microbial communities can contain hundreds of different species.

Several metabolic reconstruction tools are currently available, each offering different degrees of trade-off between automation and human intervention (20–25) (see Supplementary Table S1 for a detailed comparison). These tools follow a bottom-up reconstruction approach consisting of the following main steps: (i) annotate genes with metabolic functions; (ii) retrieve the respective biochemical reactions from a reaction database, such as KEGG (26); (iii) assemble a draft metabolic network; (iv) manually curate the draft model. The last step includes several tasks, such as adding missing reactions required to generate biomass precursors (gap-filling), correcting elemental balance and directionality of reactions, detecting futile cycles, and removing blocked reactions and dead-end metabolites (see (27) for a detailed protocol). If these problems are not resolved, the model can generate unrealistic phenotype predictions, such as incorrect biomass yields, excessive ATP generation, false gene essentiality, or incorrect nutritional requirements. The manual curation step is time-consuming and includes repetitive tasks that must be performed for every new reconstruction.

**Figure 1.**
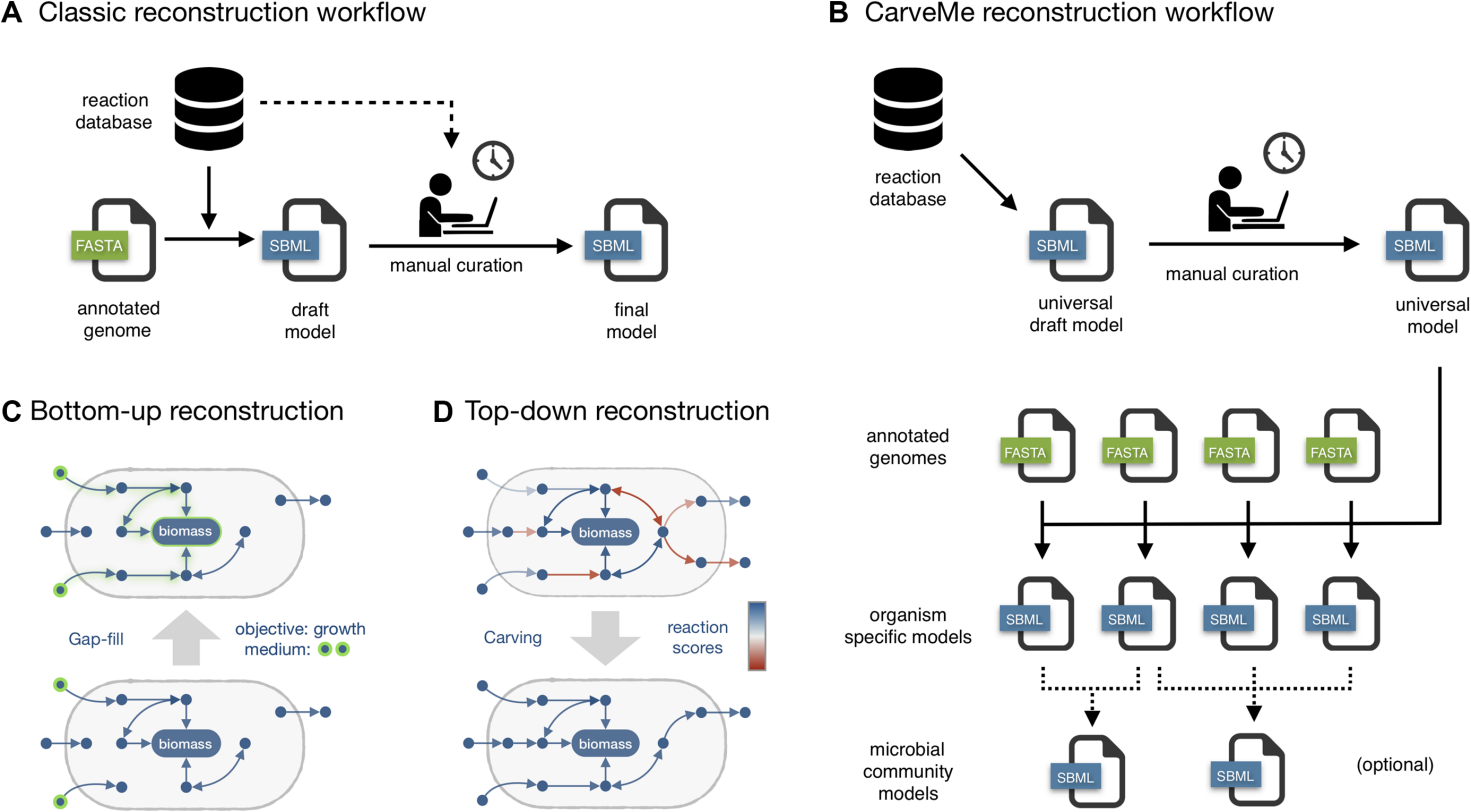
Comparison between bottom-up and top-down genome-scale metabolic model reconstruction: (**A**) in the traditional (bottom-up) approach, a draft model is automatically generated from the genome of a given organism (by homology/orthology prediction against annotated genes), followed by extensive manual curation; (**B**) in the top-down approach, a universal model is automatically generated and manually curated. This model is then used as a template for organism-specific model generation (*carving*), a process that identifies (by homology/orthology predictions) which reactions are present in the given organism. This process does not require manual intervention and can be easily parallelizable to automatically generate a large number of models. Optionally, this can be also applied to the generation of microbial community models by merging single-species models; (**C**) during bottom-up reconstruction, new reactions are iteratively added to the network for gap-filling purposes. This process is context dependent, i.e. it requires specifying the environmental conditions (growth medium) and the expected phenotype (usually biomass formation); (**D**) top-down reconstruction is a context-independent process that infers gapless pathways from genetic evidence alone by assigning confidence scores to all the reactions in a universal model.

In this work, we present CarveMe, a new reconstruction tool that shifts this paradigm by implementing a top-down reconstruction approach (Figure 1). We begin by reconstructing a universal metabolic model, which is manually curated for the common problems mentioned above. Notably, this universal model is simulation-ready: it includes import/export reactions, a universal biomass equation, and contains no blocked or unbalanced reactions. Subsequently, for every new reconstruction, the universal model is converted to an organism-specific model using a process called ‘carving’ (see Materials and Methods section for details). In essence, this process removes reactions and metabolites not predicted to be present in the given organism, while preserving all the manual curation and relevant structural properties of the original model. The lack of manual intervention makes this process automatable and the reconstruction of multiple species can be easily parallelized (Figure 1B). Unlike traditional (bottom-up) gap-filling, which iteratively adds reactions to enable growth on different media (Figure 1C), the top-down approach is able to infer the uptake/secretion capabilities of an organism from genetic evidence alone (Figure 1D), making it especially suitable for organisms that cannot be cultivated under well-defined media. Finally, CarveMe automates the creation of microbial community models by merging selected sets of single-species models into community-scale networks.

## MATERIALS AND METHODS

### Universal model building

CarveMe provides a Python script to build an universal draft model of metabolism by downloading all reactions and metabolites in the BiGG database (28) into a single SBML file (BiGG version 1.3 was used during this work). All associated metadata are stored as SBML annotations. The same script assists with automated curation tasks to build a final universal model (as described next).

### Universal bacterial model

The draft model built from the entire BiGG database was manually curated to generate a fully-functional universal model of bacterial metabolism. First, all reactions and metabolites present in eukaryotic compartments were removed. An universal biomass equation was then added to the model. This equation was adapted from the *Escherichia coli* biomass composition (29) in accordance to a recent study on universal biomass components in prokaryotes (30).

Next, we constrained reaction reversibility to eliminate thermodynamically infeasible phenotypes. Lower and upper bounds for the Gibbs free energy change for each reaction (Δ*G*_*r*_) were estimated using the formula:

\begin{equation*} \Delta G_r = \Delta G_r^o + RT \ln Q \end{equation*}

where $\Delta G_r^o$ was calculated using the component contribution method (31), *R* is the universal gas constant, and the temperature *T* was set to 298.15 K (25 C). The reaction quotient (*Q*) denotes the product-to-substrate ratio, which was limited by the physiological bounds imposed on metabolite concentrations. A recent study showed that absolute metabolite concentrations are conserved among different kingdoms of life (32). The concentrations measured in this study were used as reference and allowed to vary by 10-fold with respect to the measured values. All other metabolites were allowed to vary between 0.01 and 10 mM. The reactions were set as irreversible in the thermodynamically feasible direction whenever the estimated bounds for Δ*G*_*r*_ were either strictly positive or strictly negative. Since $\Delta G_r^o$ could not be determined for all reactions, additional heuristic rules were applied: (i) ATP-consuming reactions were not allowed to proceed in the reverse direction; (ii) reactions present in thermodynamically curated models (29,33) assumed the reversibility constraints adopted in these models.

Atomically unbalanced reactions were removed from the model. If not removed, these reactions can lead to spontaneous mass generation and unrealistic yields. Subsequently, blocked reactions and dead-end metabolites were determined using flux variability analysis and removed from the model.

Finally, the model was simulated under different medium compositions, including minimal medium (M9 with glucose), and complete medium (all uptake reactions allowed to carry flux). The model was tested for biomass and ATP production. Unlimited ATP generation was detected by flux variability analysis (34) and the reversibility of the reactions involved in energy-generating cycles was manually constrained in an iterative way until all reactions operated in the most thermodynamically favorable direction.

### Specialized templates

The universal biomass composition does not contain membrane and cell wall components specific for Gram-negative and Gram-positive bacteria, which can lead to false negative gene essentiality predictions for lipid biosynthesis pathways that generate these membrane/cell wall precursors. We generated specialized templates for Gram-positive and Gram-negative bacteria by adding these components to the respective biomass composition. The Gram-positive template includes glycerol teichoic acids, lipoteichoic acids and a peptidoglycan unit. The Gram-negative template includes phosphatidylethanolamines, murein and a lipopolysaccharide unit. We also provide a template for cyanobacteria that contains a thylakoid compartment for photosynthesis, and a template for archaea that contains ether lipids in the cell membrane composition and lacks peptidoglycans.

In all cases, the final biomass composition is normalized to represent 1 gram of cell dry weight. During reconstruction, the user can select the universal bacterial template or one of the specialized templates. Furthermore, a utility to automatically generate customized templates with user-provided biomass compositions is included.

### Gene annotation

The amino acid sequences for all genes in the BiGG database were downloaded from NCBI and stored as a single FASTA file. This file is used to align the input genome files given by the user (using DIAMOND (35)) and find the corresponding homologous genes in the BiGG database. The user can provide the genome as a DNA or protein FASTA file (note that raw genome files are not supported, since open reading frame identification is outside the scope of the tool).

Alternatively, the user can provide an alignment file externally generated with eggnog-mapper (36), which provides higher confidence for functional annotation by refining the alignments with orthology (rather than homology) predictions.

### Reaction scoring

The alignment scores obtained in the previous step are mapped to reaction scores using gene-protein-reaction (GPR) associations (this process is illustrated in Supplementary Figure S8). The goal is to score the certainty that a reaction is present in the given organism. Gene scores are first converted to protein scores as the minimum score of all subunits that form a protein complex. Protein scores are then converted to reaction scores by summing the scores of all isozymes that can catalyze a given reaction. Customized GPR associations for the reconstructed organism are generated during this process.

The final scores are normalized to a median value of 1 and typically follow a log-normal distribution. Enzyme-catalyzed reactions without genetic evidence are given a negative score (default: –1), and spontaneous reactions are given a neutral score.

### Model carving

Carving is the process of converting a universal model into an organism-specific model by removing reactions and metabolites unlikely to be present in the given organism. This is performed by solving a mixed integer linear program (MILP) that maximizes the presence of high-score reactions and minimizes the presence of low-score reactions while enforcing network connectivity (i.e. gapless pathways):

\begin{equation*} \begin{aligned} \max & \, s^T (y^f + y^r) \\ \text{s.t.} & \\ & S \cdot v = 0 \\ & v >-M y^r + \varepsilon y^f \\ & v < - \varepsilon y^r + M y^f \\ & v_i >0 \quad \forall i \in \lbrace \text{forward irreversible}\rbrace \\ & v_i < 0 \quad \forall i \in \lbrace \text{backward irreversible}\rbrace \\ & y^r + y^f \le 1 \\ & y^r, y^f \in \lbrace 0,1\rbrace ^n \\ & v_\text{growth} >v_\text{growth}^\text{min} \end{aligned} \end{equation*}

where *s* is the reaction scores vector (determined in the previous step), *v* is the flux vector, *y*^*f*^ and *y*^*r*^ are binary vectors indicating the presence of flux in the forward or backward reaction, *S* is the stoichiometric matrix, ϵ is the minimum flux carried by an active reaction (default: 0.001 mmol/gDW/h), *M* is a maximum flux bound (default: 100 mmol/gDW/h), and $v_\text{growth}^\text{min}$ is the minimum growth rate (default: 0.1 h^–1^).

After solving the MILP problem, all inactive reactions are removed from the model, including all consequently orphaned genes and metabolites. The final model is then exported as an SBML file. The user can select between the latest (FBC2) or the legacy (COBRA) version.

### Ensemble generation

CarveMe allows the generation of ensemble models. This is performed by randomizing the weighting factors of reactions without genetic evidence, and solving the MILP problem multiple times to generate alternative models. The size of the ensemble (number of models) is selected by the user, and the final ensemble is exported as a single SBML file.

For the purpose of phenotype array simulation and gene essentiality prediction, a voting threshold (*T*) is used to determine a positive outcome (i.e. a substrate is growth-supporting or a gene is considered essential, if the percentage of models that agree with such phenotype is larger than *T*). Simulation results with multiple thresholds (10%, 50%, 90%) are reported in this work.

### Experimental constraints

Experimental data can be provided as additional input during reconstruction in a tabular format. These can be used to indicate the presence, absence, or preferred direction of a given set of reactions (including intracellular reactions and metabolite exchange reactions). According to the level of confidence, these can be provided as ‘hard’ or ‘soft’ constraints.

*Soft* constraints are specified as a mapping from reactions to one of three possible values that modify the MILP objective, giving a different weight to the binary variables associated with the respective reactions: 1) forward direction preferred; –1) backward direction preferred; 0) reaction should not occur.

*Hard* constraints are simply a list of flux bounds ($v_i^\text{min} < v_i < v_i^\text{max}$). They can be used to force or block the utilization of any given reaction (please note that they can also make the MILP problem infeasible so they should be used with care).

### Gap-filling

The user can (optionally) provide a list of media where the organism is expected to grow. If the model does not reproduce growth on these media after reconstruction, CarveMe will perform additional gap-filling to enable the expected phenotypes. The implementation is similar to bottom-up gap-filling methods, except that the reactions scores previously calculated are used as weighting factors. This increases the probability that a pathway with some level of genetic evidence is selected over an alternative pathway with lower evidence. The problem is formulated as follows:

\begin{equation*} \begin{aligned} \min & \, \sum _{i \in R} \left(\frac{1}{1+s_i}\right)y_i \\ \text{s.t.} & \\ & S \cdot v = 0 \\ & lb < v < ub \\ & y_i lb_i < v_i < y_i ub_i \quad \forall i \in R\\ & y_i \in \lbrace 0,1\rbrace \quad \forall i \in R \\ & v_\text{growth} >v_\text{growth}^\text{min} \end{aligned} \end{equation*}

where *R* is the set of reactions in the universe not present in the model, *s*_*i*_ is the annotation score of reaction *i* (0 for reactions without score), *lb* and *ub* are the lower and upper flux bound vectors, and $v_\text{growth}^\text{min}$ is the same as previously defined.

### Microbial community models

CarveMe provides a script to merge a selected set of single-species models into a microbial community model. The result is an SBML model where each species is assigned to its own compartment and the extracellular environment is shared between all species. Several options are provided, such as the creation of a common community biomass reaction, creation of isolated extracellular compartments connected by an external metabolite pool, and the initialization of the community environment with a selected growth medium. The model can be readily used for flux balance analysis (just like a single-species model) to explore the solution space of the community phenotype, and also for the application of community-specific simulation methods (10,11).

### Simulation of phenotype arrays

Simulation of phenotype arrays was performed by constraining the respective models to M9 minimal medium (with a maximum uptake rate of 10 mmol/gDW/h for every compound). For each type of array (carbon, nitrogen, sulfur, phosphorus), the default source of the given element (respectively: glucose, ammonia, sulfate, phosphate) was iteratively replaced with the respective compounds in the array. For *Shewanella oneidensis* the default carbon source was dl-lactate. A phenotype was considered viable if the growth rate was at least 0.01 h^–1^. The experimental data used for validation was obtained from (33,37–40).

### Determination of gene essentiality

Gene essentiality was determined by iteratively evaluating the impact of single gene deletions using the respective gene-protein-reaction associations. For each organism, the evaluation is restricted to the set of genes common to all the models. The media compositions were defined as M9 minimal medium (with glucose) for *E. coli*, M9 (with succinate) for *Pseudomonas aeruginosa*, LB medium for *Bacillus subtilis* and *S. oneidensis*, and complete medium for *Mycoplasma genitalium*. The experimental data used for validation was obtained from (29,37,40–42).

### Performance metrics

The performance metrics used to evaluate the phenotype array simulations and gene essentiality predictions were defined as follows:

\begin{equation*} \begin{aligned} \text{Precision}: & \quad \text{TP} / (\text{TP} + \text{FP}) \\ \text{Sensitivity}: & \quad \text{TP} / (\text{TP} + \text{FN}) \\ \text{Specificity}: & \quad \text{TN} / (\text{TN} + \text{FP}) \\ \text{Accuracy}: & \quad (\text{TP} + \text{TN}) / (\text{TP} + \text{FP} + \text{FN} + \text{FN}) \\ F_1\text{-score}: & \quad 2 \text{ TP} / (2 \text{ TP} + \text{FN} + \text{FP}) \\ \end{aligned} \end{equation*}

where the true positive (TP), false positive (FP), true negative (TN) and false negative (FN) cases were determined as described above.

### Human gut bacterial species

The reconstructions were performed using the genome sequences and growth media provided in (43). The uptake/secretion data collected by (44) were provided as soft constraints during reconstruction (note that these data are reported at species, rather than strain level, hence the confidence level is low). Oxygen preferences were extracted from the PATRIC database (45) and used as hard constraints. Each model was gap-filled for the subset of media conditions where growth was observed.

### Technical details

CarveMe is implemented in Python 2.7. It requires the *framed* python package (version 0.4) for metabolic modeling, which provides an interface to common solvers (Gurobi, CPLEX), and import/export of SBML files through the libSBML API (46). In this work, all MILP problems were solved using the IBM ILOG CPLEX Optimizer (version 12.7).

## RESULTS

### Universal model of bacterial metabolism

We built an universal model of bacterial metabolism by downloading reaction data from BiGG (accessed: version 1.3) (28), a database that integrates data from 79 genome-scale metabolic reconstructions (23 unique species). Although BiGG includes a few eukaryotic reconstructions (such as yeast, mouse, and human), prokaryotes, especially bacteria, are better represented, including species from 14 different genera. The universal model contains all BiGG reactions with the exception of those exclusive to eukaryotic organisms. The model underwent several curation steps (see Methods), including estimation of thermodynamics, verification of elemental balance, elimination of energy-generating cycles, and integration of a core biomass composition. The final model includes three compartments (cytosol, periplasm and extracellular), 2383 metabolites (representing 1503 unique compounds) and 4383 reactions (2463 enzymatic reactions, 1387 transporters and 473 metabolite exchanges). From this universal model, we derived four specialized templates (Gram-positive and Gram-negative bacteria, archaea and cyanobacteria). These templates contain modified biomass equations to account for the respective differences in cell wall/membrane composition. The template for cyanobacteria additionally includes a thylakoid compartment. During reconstruction, the user can select between the generic or specialized (or even a user-provided) universal template. In addition to the universal model, we generate a sequence database with a total of 30 814 unique protein sequences derived from the gene–protein–reaction associations of the original models. These are used to align the input genomes and obtain confidence scores for the respective reactions in the universal model.

### Comparison with manually curated metabolic models

The quality of the models generated with CarveMe was evaluated regarding their ability to reproduce experimental data and compared to previously published manually curated models. Two reference model organisms were selected as case-studies, *E. coli* (strain K-12 MG1655) and *B. subtilis* (strain 168). These are the most well-studied Gram-negative and Gram-positive bacteria, respectively, with highly-curated metabolic models. We also selected four organisms that are not part of the BiGG database, namely *M. genitalium* (strain G-37), *P. aeruginosa* (strain PA01), *R. solenacearum* (strain GMI1000) and *S. oneidensis* (strain MR-1).

**Figure 2.**
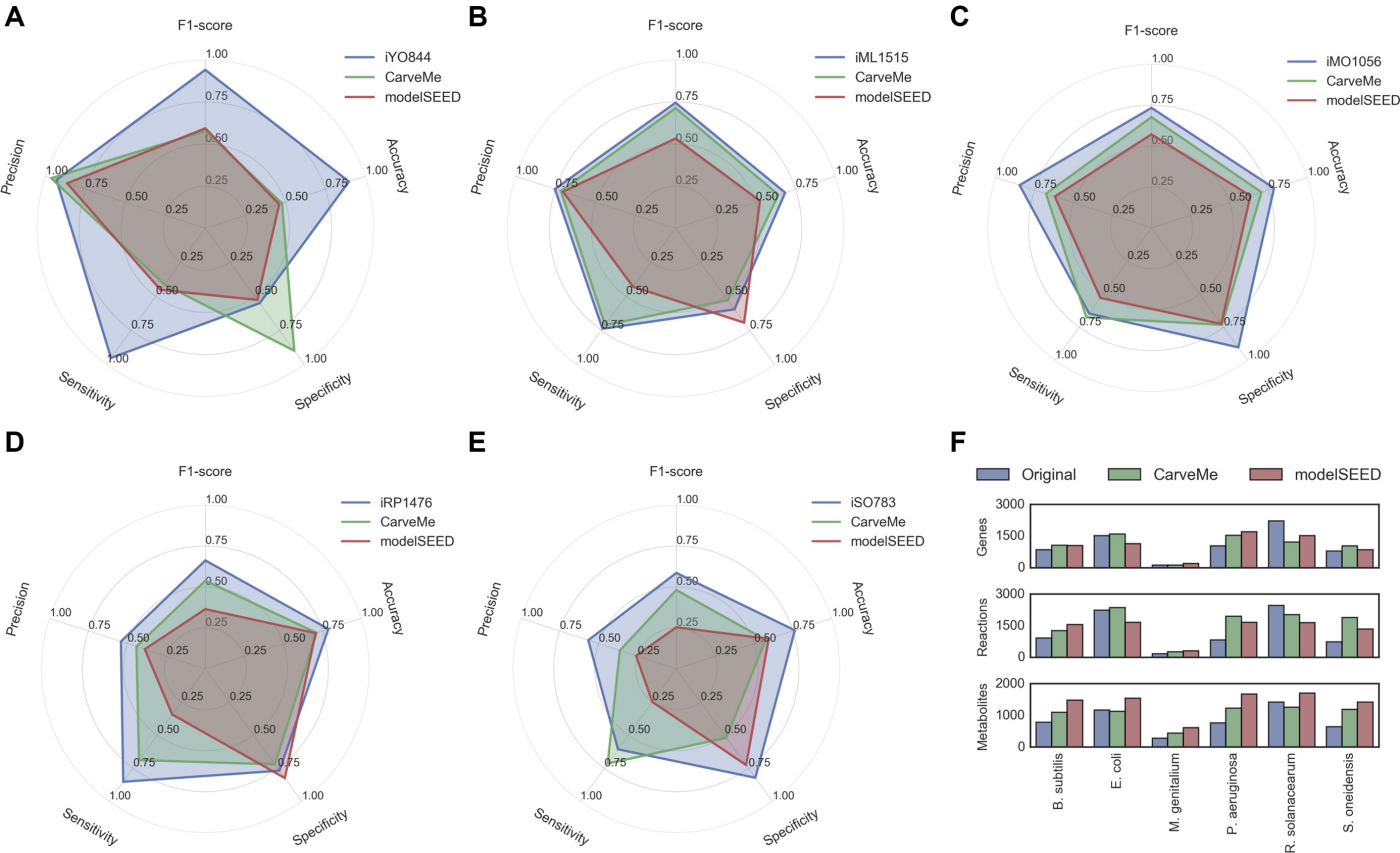
Model summary and phenotype array simulation results: (A–E) simulation results for (**A**) *B. subtilis*, (**B**) *E. coli*, (**C**) *P. aeruginosa*, (**D**) *R. solanacearum* and (**E**) *S. oneidensis*. (**F**) Summary of the 18 reconstructions analyzed in this study with regard to number of genes, reactions (only gene-associated reactions considered) and metabolites (all unique compounds).

The genomes of these strains were downloaded from NCBI RefSeq (release 84) (47) and used to build models with CarveMe. The manually curated models were taken from their respective publications (37–39,48–50). Furthermore, to analyze how CarveMe performs in comparison to other automated reconstruction tools, the same genomes were used to generate genome-scale models using the modelSEED pipeline (https://kbase.us/) (21). Figure 2F shows a summary of all models in terms of total number of genes (enzyme-associated) reactions and metabolites.

All the models were used to simulate substrate utilization (Biolog phenotype arrays) and gene essentiality, and the results were systematically compared against published experimental data. Two exceptions were *M. genitalium* (no Biolog data available) and *R. solanacearum* (no gene essentiality data available). The predictive ability of the models was evaluated in terms of multiple metrics: accuracy, precision, sensitivity, specificity, and F1-score (see Methods for details on the experimental setup, metrics description, and data sources).

Notably, four out of five models generated with CarveMe were able to reproduce growth on minimal media from genome data alone, without specifying any growth requirements during reconstruction or performing any additional gap-filling after reconstruction. The other model (*R. solanacearum*) required one single gap-filling reaction (asparagine synthetase) to reproduce growth on minimal medium. All the models generated with modelSEED required the specification of the minimal media during reconstruction. Without this *a priori* information, the models were unable to reproduce growth on the given media.

#### Benchmark 1: Biolog phenotype arrays

Regarding substrate utilization, it can be observed that the performance of CarveMe models is in-between the performance of manually curated models and those generated with modelSEED (Figure 2). In some cases the modelSEED models display higher specificity (especially for *S. oneidensis*), but perform worse on other metrics. For *E. coli*, the CarveMe model performs very closely to the iML1515 model, outcompeting the modelSEED model. In the case of *B. subtilis*, both CarveMe and modelSEED perform considerably worse than the iYO844 model. This difference in performance can be explained by the lack of annotated transporters for *B. subtilis*, which have been manually added in the curated model.

To test the influence of transporter annotation, we repeated the simulations for all models, this time allowing free diffusion of any metabolites without associated transporters (Supplementary Figure S1). It can be observed that for *B. subtilis* the automated reconstructions now perform more closely to the manually curated model, with the modelSEED reconstruction outperforming CarveMe. For the other organisms, one can also observe a smaller performance gap between the manually curated models and the automatic reconstructions, with CarveMe generally outperforming modelSEED.

#### Benchmark 2: Gene essentiality

**Figure 3.**
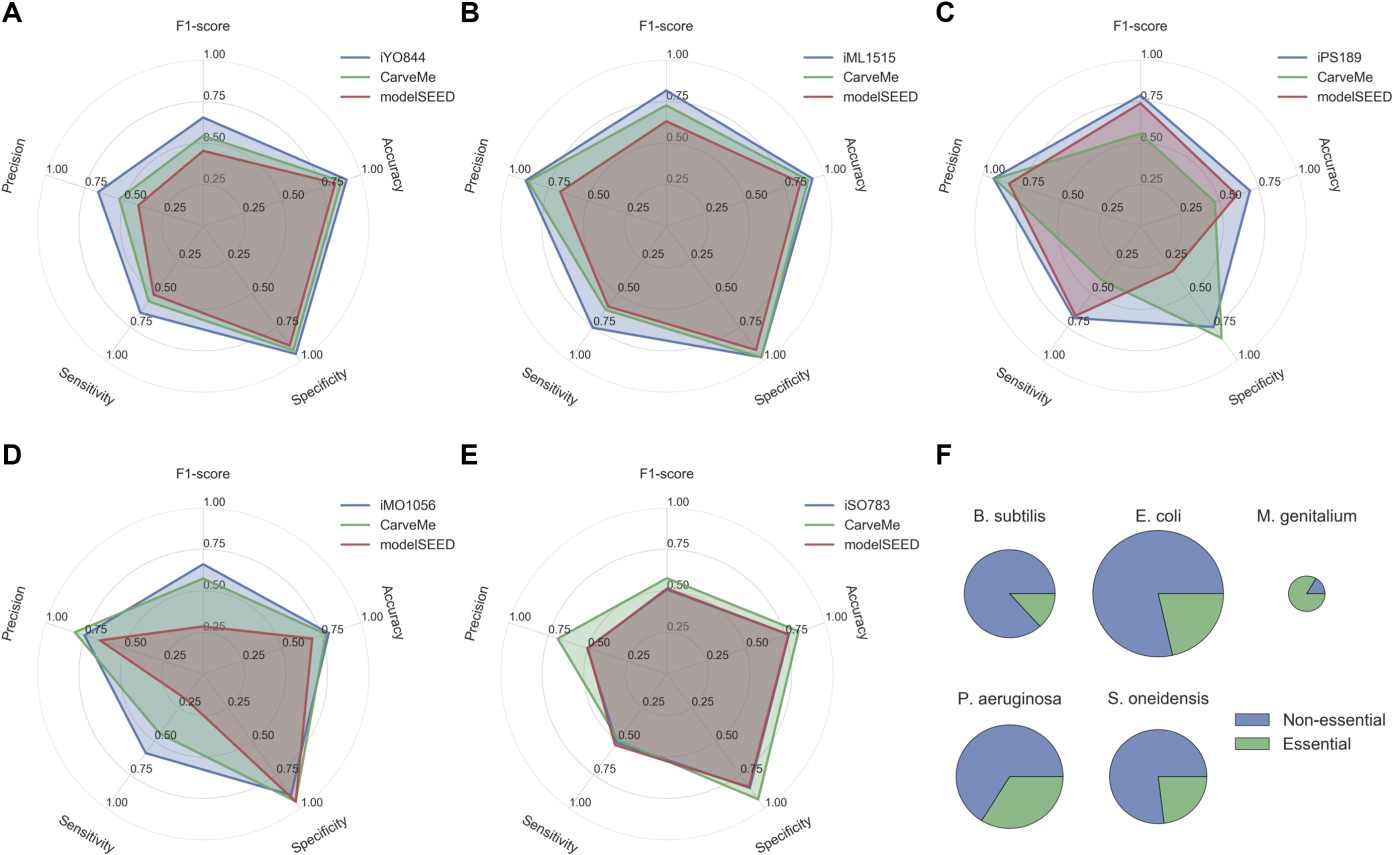
Model Gene essentiality results: (A-E) Gene essentiality results for (A) *B. subtilis*, (B) *E. coli*, (C) *M. genitalium*, (D) *P. aeruginosa*, and(E) *S. oneidensis*; (F)Fraction of essential genes per organism (the circle area is proportional to the number of metabolic genes common to all the models).

Regarding the ability to predict gene essentiality, the same general pattern can be observed, with CarveMe models displaying a performance in-between the manually curated models and modelSEED (Figure 3). Two exceptions are *M. genitalium* (modelSEED outperforms CarveMe for most metrics) and *S. oneidensis* (iSO783 and modelSEED display similar performance, being both outperformed by CarveMe).

One common pattern for most CarveMe models (and modelSEED to some extent) is a slightly decreased sensitivity with regard to gene essentiality (i.e. many essential genes reported as false negatives) when compared to the manually curated models. One possible reason is the utilization of generic biomass templates that exclude non-universal cofactors (30) and other organism-specific biomass precursors. To test the influence of biomass composition on gene essentiality prediction, we built models for *E. coli* and *B. subtilis* using three distinct biomass compositions: our universal (core) biomass compositon, our Gram-negative and Gram-positive compositions, and the organism-specific compositions taken directly from the manually curated models (Supplementary Figure S2). Confirming to our hypothesis, we observed a gain in sensitivity (and consequently in the F1-score) when using organism-specific biomass compositions. Nonetheless, the specialized Gram-negative and Gram-positive compositions perform closer to the organism-specific compositions than to the core biomass composition. Hence, the utilization of specialized templates seems to be a suitable compromise when an organism-specific biomass composition is not available.

#### Benchmark 3: Ensemble modeling

A general limitation in building genome-scale metabolic models, given the available data, is the existence of several equally plausible models. This has been explored by Biggs and Papin (51), showing that when gap-filling a model for multiple growth media, the selected order of the media influences the final network structure. Another source of ambiguity is the degeneracy of solutions when solving an optimization problem to generate the model itself. To tackle this issue, CarveMe allows the generation of model ensembles. The user can select the desired ensemble size, and then use the ensemble model for simulation purposes in order to explore alternative solutions.

We generated model ensembles (*N* = 100) for all organisms and repeated the phenotype array simulations and gene essentiality predictions using different voting thresholds (10%, 50%, 90%) to determine a positive prediction (see Methods). To confirm that the ensemble generation is unbiased, we calculated the Jaccard distance between every pair of models in each ensemble (Supplementary Figure S3A). We observed that all ensembles show some extent of variability with the pairwise distances following normal distributions. The ensemble for *M. genitalum* shows the highest variability (average distance of 0.48), whereas the *E. coli* ensemble shows the lowest variability (average distance of 0.04). This distance is a reflection of the degree of uncertainty in the network structure.

Interestingly, we observe that the variability in network structure does not necessarily reflect into phenotype variability, with the different models within an ensemble often predicting the same phenotypes. Regarding the phenotype array simulations (Supplementary Figure S3B–F), we can generally observe an increase in sensitivity at the cost of decreased specificity with respect to the single model reconstructions. With regard to gene essentiality (Supplementary Figure S3G–K), there are no observable differences between the ensemble model simulations and the results obtained with single models. This result is most likely a reflection of the robustness of cellular function to perturbations in the network structure during evolution (52,53). In both scenarios, the results seem to be independent of the voting threshold (with exception of the 90% threshold, where often no positive consensus is obtained).

### Metabolic models for the human gut bacteria

The human gut microbiome is of particular interest for metabolic modeling due to its impact on human health (54–56). In this regard, one major hurdle is the characterization of gut bacterial species in terms of growth requirements and metabolic potential. In a recent study from our group, the growth of 96 phylogenetically diverse gut bacteria was characterized across 19 different media (15 defined and 4 complex media compositions) (43). From this list, a total of 47 bacteria are also included in the AGORA collection, a recently published collection of 773 semi-manually curated models of human gut bacteria (57). These models, however, could not reproduce the metabolic needs experimentally observed by Tramontano *et al*., highlighting the need for the use of validated growth media for species that are distant from model organisms.

In this work, we used CarveMe to reconstruct the metabolism of 74 bacterial strains that grew in at least one defined medium in the Tramontano *et al.* study. Since CarveMe allows users to provide experimental data in tabular format during reconstruction, we used a recently published collection of literature-curated uptake/secretion data for human gut species (44) to further refine the reconstructed models (see Materials and Methods). The resulting models thus represent an up-to-date and experimentally-backed collection for gut bacteria.

To understand how the media information contributed to the model refinement, we analysed the gap-filling reactions introduced in each model when these data is provided during reconstruction. Interestingly, we observed that the total number of gap-filling reactions is negatively correlated with the genome size of the respective organism (Pearson’s *r* = –0.29, *P* = 0.0055; Supplementary Figure S4A). Considering that small-genome species usually tend to harbor many auxotrophies, and also that many of the gap-filling reactions occur in amino acid metabolism, it is likely that such auxotrophies are not correctly identified due to poor annotation of the transport-associated genes.

Finally, we compared the number of gap-filled reactions between CarveMe and AGORA models (40 models in common) and observed that they are well correlated (Pearson’s *r* = 0.5, *P* = 0.0047; Supplementary Figure S4B). Interestingly, the most extensively gap-filled organism in both collections is *Bifidobacterium animalis* (subsp. lactis Bi-07), which is also the organism with smaller number of genes. The most extensively gap-filled subsystem across all organisms, ‘Cofactor and Prosthetic Group Biosynthesis’, is also the same for both collections. Overall, this shows that the quality of the CarveMe models (even without the Tramontano *et al*. data) is comparable to the manually-curated AGORA collection, which further highlights the advantages of our top-down curation approach.

### Large-scale model reconstruction and community models

To demonstrate the scalability of CarveMe, and to provide the research community with a collection of reconstructed models spanning a wide variety of microbes, we reconstructed 5587 bacterial genome-scale models. These correspond to all bacterial genomes available in NCBI RefSeq (release 84) (47) that are classified as reference or representative assemblies at the strain level. This collection of models represents metabolism across all (currently sequenced) bacterial life and thus can help uncovering principles underlying the architecture and diversity of metabolic networks.

**Figure 4.**
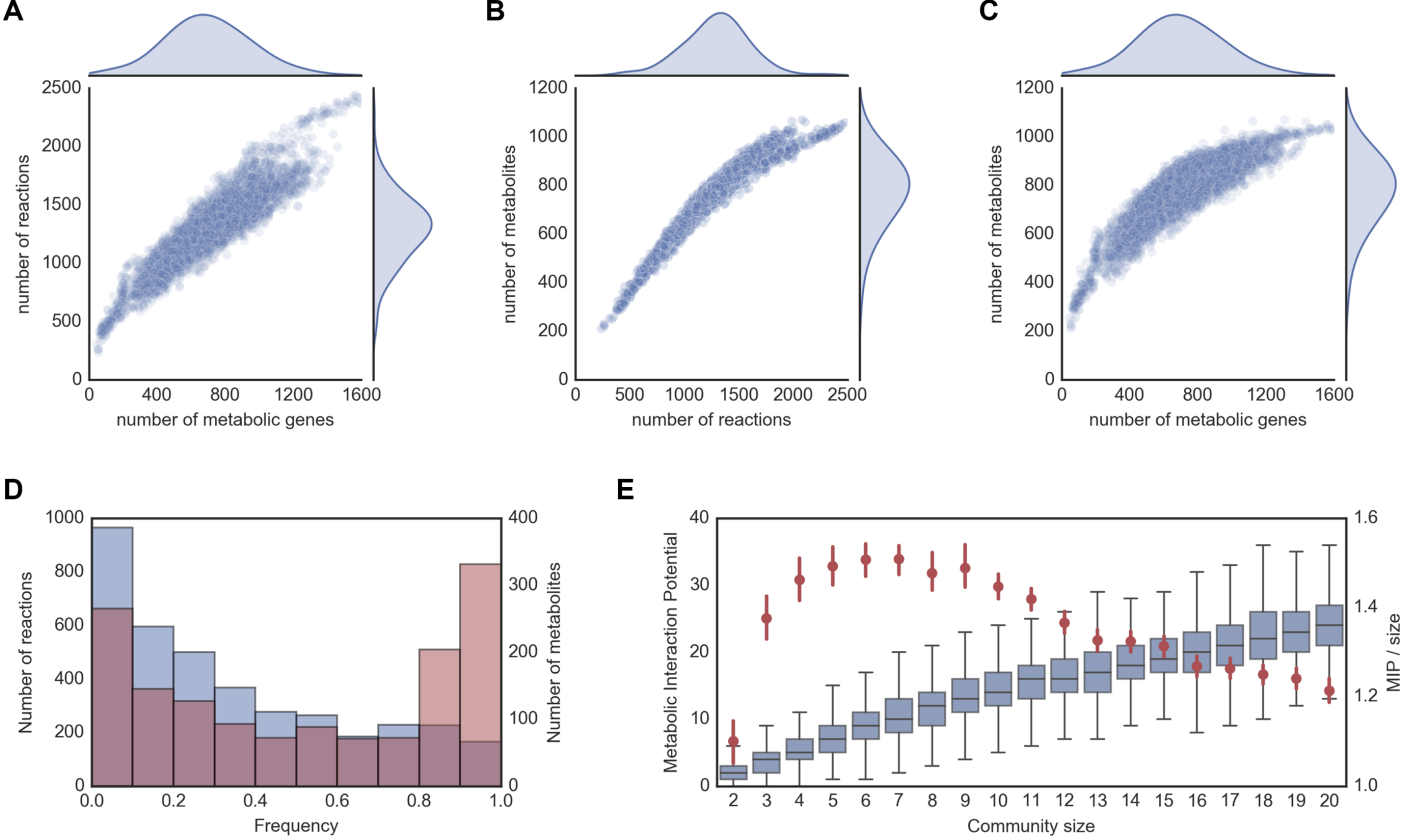
NCBI RefSeq reconstruction collection summary: (A) Number of genes vs number of reactions per organism; (B) Number of reactions vs number of metabolites per organism; (C) Number of genes vs number of metabolites per organism; (D) Reaction and metabolite frequency distribution across all organisms; (E) Metabolic interaction potential (MIP) of randomly assembled communities of different sizes, including absolute MIP values (box plots), and average MIP normalized by community size (red dots with 95% confidence intervals).

Here, we analyse the distribution of the number of (annotated) metabolic genes, reactions and metabolites across all organisms (Figure 4). It is possible to observe that these are all normally distributed, with the average organism containing 691 metabolic genes, 1308 reactions and 792 metabolites. The smaller metabolic networks belong to the *Mycoplasma* genus with as few as 238 reactions (*Mycoplasma ovis* str Michigan), whereas the larger metabolic networks belong to the *Klebsiella* and *Escherichia* genera, with up to 2472 reactions (*Klebsiella oxytoca* str CAV1374). We note that these numbers are indicative, as they can be biased or restricted by the quality of the gene annotation and the scope of the reaction database.

Interestingly, as the size of the metabolic networks grows, there there is an asymptotic trend of the number of metabolites with respect to the number of genes and reactions (Figure 4B and C). This indicates a saturation of metabolite space relative to the expansion of the enzyme and reaction space. This can occur due to, for example, alternative pathways acting on the same metabolites. In fact, earlier studies show that evolution favors the selection of specialized enzymes that co-exist with ancestral promiscuous enzymes, as well as differentially regulated isoforms, working as a control mechanism that confers robustness towards environmental and genetic perturbations (58–60).

We further looked at how frequently individual reactions and metabolites occur across species (Figure 4D). The frequency of reactions shows a negative exponential distribution with 30% of all reactions being present in less than 10% of the organisms, and only 5% of reactions being present more than 90% of the organisms. Interestingly, the metabolite frequency shows instead a bimodal distribution with peaks below 10% frequency (≈ 20% metabolites) and above 90% frequency (≈25% metabolites). As expected, the high frequency reactions and metabolites are distributed across the primary metabolism, including carbohydrate, lipid, nucleotide, amino acid and energy metabolism (Supplementary Figure S5).

Next, we illustrate the use of our model collection to build microbial community models and explore inter-species interactions. We created random assemblies of microbial communities of different sizes (up to 20 member species per community, 1000 assemblies for each community size) and calculated their metabolic interaction potential (MIP score, as defined in (11)). In brief, the MIP score of a community is a measure of the number of compounds that can be exchanged between the community members, allowing the community to reduce its dependence on the environmental supply of nutrients.

The results obtained with this collection (Figure 4E) closely match those previously obtained with a smaller model collection (1503 bacterial strains, generated with modelSEED) (11). In particular, while the potential for interaction increases with the community size, the number of interactions normalized by the community size shows a maximum at a relatively small community size (6–7 species).

## DISCUSSION

We introduced CarveMe, an automated reconstruction tool for genome-scale metabolic models that implements a novel top-down reconstruction approach. Our extensive benchmark shows that the performance of CarveMe models, in terms of reproducing experimental phenotypes, is comparable to that of manually curated models and often exceeds the performance of models generated with modelSEED. Notably, many of our models correctly reproduced growth on minimal media without any growth requirements being specified during reconstruction. This was not observed with modelSEED, making CarveMe especially suited for situations where the growth requirements are not known *a priori* (e.g. microbes that cannot be cultivated under well-defined media). In case a generated model is not able to reproduce growth on a given medium, CarveMe can additionally perform gap-filling to enable the expected phenotype. The implemented gap-filling approach differs from methods that simply minimize the number of added reactions (61), by prioritizing reactions according to their gene homology scores, similarly to the likelihood-based method proposed by Benedict and co-workers (62). Note that our approach differs from the latter in the sense that the gap-filling is applied to a model that was top-down generated from a fully-functional universal model.

We used the BiGG database (28) to build our universal model, which offers some advantages compared to other commonly used reaction databases such as KEGG, Rhea, or modelSEED (21,26,63). First, it was built by merging several genome-scale reconstructions (ranging from bacterial to human models), which leverages on the curation efforts that were applied to these models. Secondly, BiGG reactions contain gene-protein-reaction associations, compartment assignments, and human-readable identifiers. These aspects facilitate the reconstruction process and the utilization of the generated models. However, BiGG is limited in size and scope compared to other databases, which may result in lack of coverage of certain metabolic functions. A comparison between the reaction contents of BiGG, modelSEED and KEGG (Supplementary Figure S6), shows that BiGG contains a large number of reactions which are not present in the other databases (most likely a consequence of the manual curation efforts), but lags behind in terms of total coverage of EC numbers. We extracted all KEGG cross-references from the BiGG database and mapped them into KEGG’s global pathway map (Supplementary Figure S7). As expected, primary metabolism is essentially complete, whereas peripheral pathways associated with secondary metabolism contain multiple gaps. This makes CarveMe models suitable for common types of analysis (prediction of gene essentiality, nutritional requirements, cross-feeding interactions), while applications concerning secondary metabolism will require additional curation. Transport reactions are also often missing from most databases due to the lack of functional annotation of transporter genes (64), which can hamper the correct prediction of metabolic exchanges. To cope with these issues, in future releases we plan to facilitate the integration of reaction data from multiple databases. Cofactor utilization (e.g.: NAD versus NADP preference) is another common issue in model reconstruction, being especially relevant for metabolic engineering applications where cofactor balancing is critical (65). Unlike functional annotation, cofactor preference cannot be directly predicted by homology, and methods based on structural motifs have been proposed (66,67). Although we currently do not implement such methods as part of CarveMe (mainly because their predictive power remains to be sufficiently validated), their outputs can be provided to the reconstruction pipeline as additional (generic) constraints (see Materials and Methods). Note that, despite all the limitations mentioned above, CarveMe showed high predictive performance even for organisms which are not part of the BiGG database.

The quality of the universal model used for top-down reconstruction is of paramount importance. There is a tradeoff between the desired broadness of this “universal” model and the amount of curation effort required. In this work, we opted to provide a well-curated universal model of bacterial metabolism, which can be generally used for reconstruction of any bacterial species. We also provide more refined template models (Gram-positive and Gram-negative bacteria, cyanobacteria and archaea), which account for differences in membrane/cell wall composition. In future releases, we aim to provide a larger collection of universal models in order to improve coverage of all domains of microbial life (such as yeasts and other eukaryotes).

The universal model and, consequently, all generated models are pre-curated for common structural and biochemical inconsistencies and can be readily used for simulation. Nonetheless, these models should still be considered as drafts subject to further refinement, as they might require organism-specific curation to reproduce certain phenotypes. The pipeline allows users to provide their own templates at any desired taxonomic level and provides methods to facilitate the creation and curation of such templates (see Materials and Methods). For instance, a user might decide to manually curate a universal model for a particular species and use it to reconstruct multiple strains of that species.

Finally, we have given particular attention to making CarveMe an easy-to-use tool. While the underlying *carving* procedure can work with genetic evidence alone (i.e. without requiring the specification of a medium composition), any collected experimental data (such as growth media, known auxotrophies, metabolic exchanges, presence/absence of reactions) can be provided in a simple tabular format as additional input for reconstruction. Also, it implements a modular architecture that facilitates modification/integration of new components. For instance, sequence alignments are performed with diamond (35) or can be extracted from the output of third-party tools (we currently support eggnog-mapper (36)). Another salient feature of CarveMe is its speed and easy parallelization. A single reconstruction required, on average, 3 min on a laptop computer (Intel Core i5 2.9 GHz). Indeed, this allowed us to rapidly create one of the largest publicly available metabolic model collections. The pipeline is available as an open-source command line tool, and can be easily installed on a personal computer as well as on a high-performance computing cluster. Several other features include direct download of genome sequences from NCBI, and automatic generation of microbial community models. This is expected to facilitate the use of genome-scale models by a broad range of researchers and cope with the increasing number of sequenced genomes publicly available.

## DATA AVAILABILITY

CarveMe is available at github.com/cdanielmachado/carveme with an open source license. All the models, data and scripts used in this work are available at github.com/cdanielmachado/carveme_paper. The database with the 5587 model collection is available at github.com/cdanielmachado/embl_gems.

## Supplementary Material

Supplementary Data
