# Supplementary material for: Fast automated reconstruction of genome-scale metabolic models for microbial species and communities
Source: Nucleic Acids Res. 2018 Jun 21;46(15):7542–53. doi: 10.1093/nar/gky537 (PMC6125623; doi:10.1093/nar/gky537)
Supplement: Supplementary Data [file gky537_supplemental_files.zip › supp_material_legends.pdf]

Supplementary Material Legends:

Supplementary Table 1: Feature comparison between CarveMe and other genome-scale metabolic model reconstruction tools.

Supplementary Figure 1: Phenotype array simulation results with free diffusion for compounds without associated transporters: a) *E. coli*; b*) B. subtilis*; c) *P. aeruginosa*; d) *R. solanacearum*; f) *S. oneidensis*.

Supplementary Figure 2: Gene essentiality simulation using different biomass compositions: a) *E. coli*; b) *B. subtilis*.

Supplementary Figure 3: Ensemble modeling results: a) Pairwise distance between all network structures within each ensemble; b-f) Phenotype array simulation results for (b*) E. coli*, (c) B. subtilis, (d) P. aeruginosa, (e) R. solanacearum, and (f) S. oneidensis; g-k) Gene essentiality simulation results for (g) E. coli, (h) B. subtilis, (i) M. genitalium, (j) P. aeruginosa, and (k) S. oneidensis. Panels b-k compare single model vs ensemble model simulations, where T denotes the voting threshold used to determine a positive result.

Supplementary Figure 4: Gap-filled reactions for human gut bacteria: a) Comparison between genome size and the number of gap-filled reactions per species for all CarveMe reconstructions; b) Comparison of the number of gap-filled reactions between CarveMe and AGORA models. Pearson correlation is indicated in both panels.

Supplementary Figure 5: Global distribution of reaction and metabolite frequency across the bacterial model reconstruction collection. The frequency of metabolites and reactions is indicated, respectively, by node and edge width and opacity. Image generated with iPath (https://pathways.embl.de).

Supplementary Figure 6: Comparison of the reaction coverage between different reaction databases: a) Total number of unique EC numbers present in each database; b) Total number of unique reactions present in each database. Data obtained from the MetaNetX database (https://www.metanetx.org).

Supplementary Figure 7: Mapping of BiGG reactions and metabolites into KEGG's global pathway map. Image generated with iPath (https://pathways.embl.de).

Supplementary Figure 8: Illustration of the reaction scoring algorithm based on gene alignment scores and propagation through Gene-Protein-Reaction (GPR) associations.
