# Supplementary material for: Fast automated reconstruction of genome-scale metabolic models for microbial species and communities
Source: Nucleic Acids Res. 2018 Jun 21;46(15):7542–53. doi: 10.1093/nar/gky537 (PMC6125623; doi:10.1093/nar/gky537)
Supplement: Supplementary Data [file gky537_supplemental_files.zip › supp_table_1.pdf]

Supplementary Table 1

Reconstruction Tool	Language / Technology	User Interface	Reaction Database	Automation Level	GPR Prediction	Thermodynamic calculation	Biomass equation	Gap Filliing	Simulation ready	Supports Eukaryotes	Reference
CarveMe	Python	CLI	BiGG	Full	Yes	Yes	Yes	Yes	Yes	No (*)	This study
Merlin	Java	GUI	KEGG (+ TCDB)	Partial	Yes	No	No	No	No	Yes	Dias 2017
CoReCo	Python	CLI	KEGG	Partial	No	No	No	Yes	Yes	Yes	Pitkänen 2014
RAVEN	Matlab	API	KEGG	Partial	No	No	No	Yes	Yes	Yes	Agren 2013
modelSEED	Web-based	Web	modelSEED	Full	Yes	Yes	Yes	Yes	Yes	No (**)	Henry 2010
GEMsystem	Perl	GUI	KEGG, BioCyc	Partial	No	No	No	Yes	No	No	Arakawa 2006
PathwayTools	Lisp	Web/API	BioCyc	Partial	Yes	Yes	Yes	Yes	Yes	Yes	Karp 2002

(*) Can be added through the creation of an Eukaryotic template

(**) Currently provides a reconstruction template for Plants

List of acronymns: Command Line Interface (CLI); Graphical User Interface (GUI); Application Programming Interface (API).
