# Supplementary figures and images for: Fast automated reconstruction of genome-scale metabolic models for microbial species and communities
Source: Nucleic Acids Res. 2018 Jun 21;46(15):7542–53. doi: 10.1093/nar/gky537 (PMC6125623; doi:10.1093/nar/gky537)

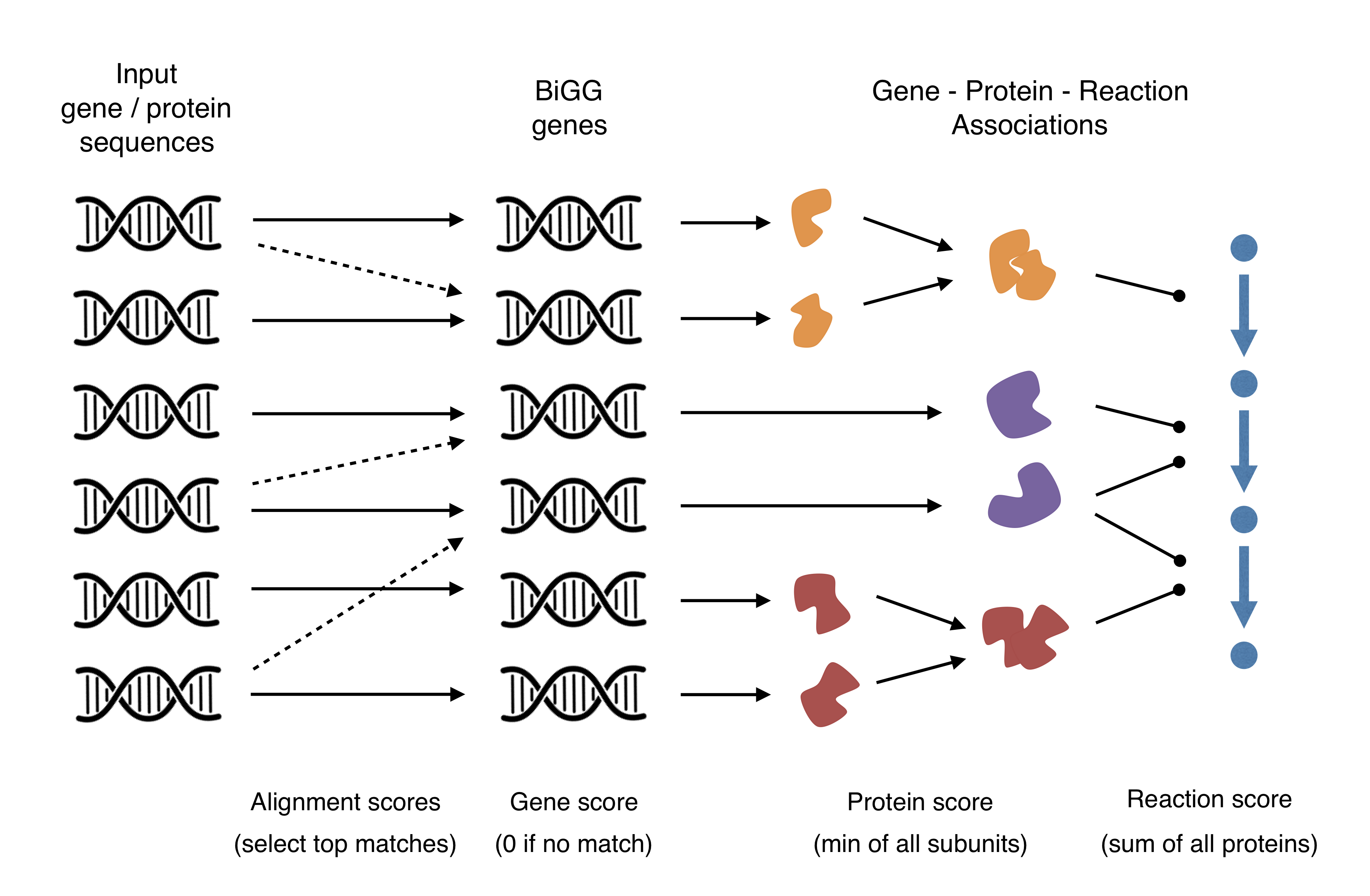

Supplement: Supplementary Data [file gky537_supplemental_files.zip › supp_fig8.png]
